# Genetic characterization of avian influenza subtype H4N6 and H4N9 from live bird market, Thailand

**DOI:** 10.1186/1743-422X-8-131

**Published:** 2011-03-21

**Authors:** Trong Wisedchanwet, Manoosak Wongphatcharachai, Supanat Boonyapisitsopa, Napawan Bunpapong, Pravina Kitikoon, Alongkorn Amonsin

**Affiliations:** 1 Emerging and Re-emerging Infectious Diseases in Animals, Research Unit, Faculty of Veterinary Science, Chulalongkorn University, Bangkok, Thailand; 2 Department of Veterinary Public Health, Faculty of Veterinary Science, Chulalongkorn University, Bangkok, Thailand; 3 Department of Pathology, Faculty of Veterinary Science, Chulalongkorn University, Bangkok, Thailand

## Abstract

A one year active surveillance program for influenza A viruses among avian species in a live-bird market (LBM) in Bangkok, Thailand was conducted in 2009. Out of 970 samples collected, influenza A virus subtypes H4N6 (n = 2) and H4N9 (n = 1) were isolated from healthy Muscovy ducks. All three viruses were characterized by whole genome sequencing with subsequent phylogenetic analysis and genetic comparison. Phylogenetic analysis of all eight viral genes showed that the viruses clustered in the Eurasian lineage of influenza A viruses. Genetic analysis showed that H4N6 and H4N9 viruses display low pathogenic avian influenza characteristics. The HA cleavage site and receptor binding sites were conserved and resembled to LPAI viruses. This study is the first to report isolation of H4N6 and H4N9 viruses from birds in LBM in Thailand and shows the genetic diversity of the viruses circulating in the LBM. In addition, co-infection of H4N6 and H4N9 in the same Muscovy duck was observed.

## Findings

Live-bird markets (LBMs) are the places where wild birds, pet birds, meat birds and domestic poultry are sold to households. In Asia including Thailand, due to the cultural preference of consuming freshly slaughtered poultry, LBMs are located in both suburban areas and center of the communities. In the markets, thousands of birds from different sources are sold in wire stacked cages containing densely packed and mixed bird populations. These conditions provide excellent environments for animal to animal and animal to human influenza virus transmissions and may result in an outbreak of influenza A virus in both animals [1,2] and humans [3,4]. Therefore, LBMs are considered a major source of influenza A virus dissemination and potential influenza A virus reassortment [5,6].

Up to date, many studies on influenza A in LBMs from various countries have been reported. During 2000-2001, 6 subtypes (9 genotypes) of low-pathogenic avian influenza (LPAI) were identified in LBMs in China [7]. Apart from Asian countries, in the US, H5N2 low pathogenic avian influenza (LPAI) viruses have been isolated from LBMs in several states in the 80 s [8]. In Thailand, only one study of influenza A viruses recovered from LBMs has ever been reported [9]. In that study, highly pathogenic avian influenza (HPAI) H5N1 viruses were isolated from both bird carcasses and healthy birds during the 2006-2007 LBM and local food market (LFM) surveillance program. The findings suggested that animal movement from H5N1 outbreak areas may introduce the virus into the markets and play an important role in emergence or re-emergence of influenza A in animals in Thailand [9]. Since LBMs play an important role in the dissemination of avian influenza virus, active surveillance of influenza A virus in LBMs is important in order to develop an early warning system and implement prevention and control strategies for influenza A outbreaks. In this study, a one year active surveillance program for influenza A viruses among avian species in LBM from Bangkok, Thailand was conducted in 2009. Influenza A subtypes, H4N6 (n = 2) and H4N9 (n = 1), were isolated from healthy Muscovy ducks. Interestingly, co-infection of H4N6 and H4N9 in the same Muscovy duck was also observed. Whole genome sequencing, phylogenetic analysis and genetic analysis of the viruses were performed. This study highlights the first LPAI subtypes H4N6 and H4N9 ever reported in poultry from Thailand.

During January to December 2009, a 12-month LBM surveillance program was carried out in LBM in Bangkok, Thailand. The LBM was visited monthly and approximately 40 samples (chicken and ducks) per market were collected at each sampling. In total, 970 samples (485 oropharyngeal and 485 cloacal swabs) were collected from 485 animals (chicken = 214, ducks = 271) from LBM located in the center of Bangkok. All swab samples were subjected to virus isolation by using embryonated egg inoculation according to WHO/OIE recommendations. Allantoic fluid from egg inoculation was tested for Hemagglutination (HA) titer using 1% chicken red blood cells. Samples testing positive for HA test were subjected for influenza A virus identification and subtyping. To identify influenza A virus, real-time-RT-PCR specific for the influenza A virus matrix (M) gene was performed as previously described [10]. To subtype influenza A virus, cDNA synthesis and RT-PCR subtyping were performed. RT-PCR was performed by using primers specific for each subtype of influenza A virus [11,12]. In this study, 3 samples collected in June, 2009 were identified as influenza A virus subtype H4N6 (n = 2) and H4N9 (n = 1) while 16 samples collected in November, 2009 were identified as H10N3. On the other hand, all other samples were negative for influenza A virus. Interestingly, two influenza A subtypes, H4N6 (CU-LM1983) and H4N9 (CU-LM1984), were isolated from oropharyngeal (CU-LM1983) and cloacal (CU-LM1984) swabs of the same duck. Both H4 subtypes have never been reported in Thailand.

After subtyping, whole genome sequencing was performed by using newly designed primers for each gene of influenza A subtypes, H4N6 and H4N9. The whole genome sequences of influenza A virus subtypes H4N6 (n = 2) and H4N9 (n = 1) were submitted to the GenBank database with accession numbers as follows: A/Muscovy Duck/Thailand/CU-LM1973/09(H4N6) (CY062545-52), A/Muscovy Duck/Thailand/CU-LM1983/09(H4N6)(CY062553-60), and A/Muscovy Duck/Thailand/CU-LM1984/09(H4N9)(CY062561-68).

**Figure 1:**
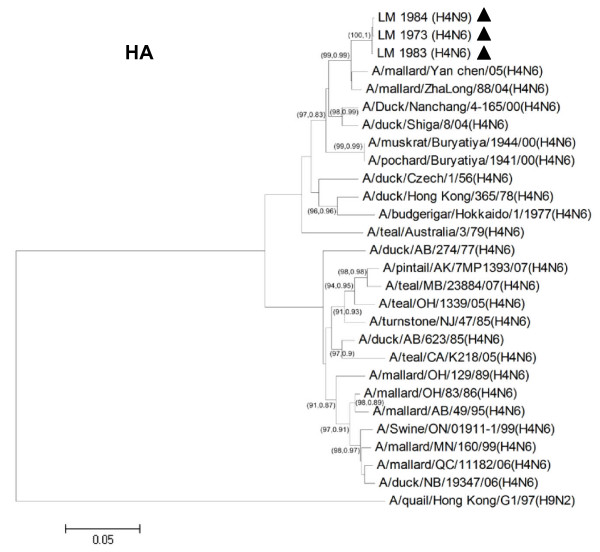
**Phylogenetic analysis of HA gene of Thai H4N6 and H4N9 viruses and other H4N6 and H4N9 influenza A viruses**. The phylogenetic tree was generated using the neighbor-joining algorithm. Bootstrap analysis with 1000 replicates and posterior probability from BMCMC analysis were performed for confirming tree topology (Bootstrap, posterior probability). The H4N6 and H4N9 influenza viruses characterized in the study are highlighted by a triangle.

**Figure 2:**
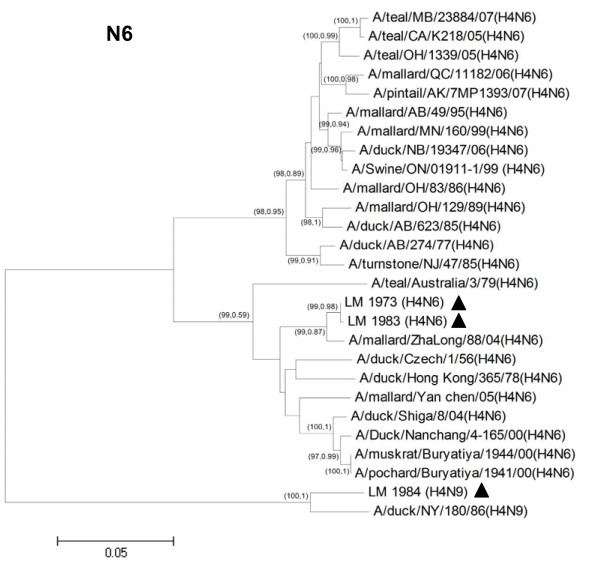
**Phylogenetic analysis of NA gene of Thai H4N6 viruses (CU-LM1973 and CU-LM1983) and other H4N6 influenza A viruses**. The phylogenetic tree was generated using the neighbor-joining algorithm. Bootstrap analysis with 1000 replicates and posterior probability from BMCMC analysis were performed for confirming tree topology (Bootstrap, posterior probability). The H4N6 and influenza viruses characterized in the study are highlighted by a triangle.

**Figure 3:**
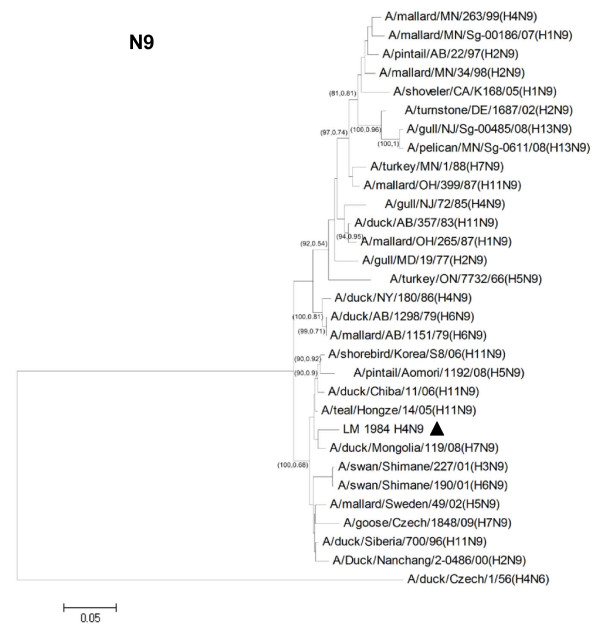
**Phylogenetic analysis of NA gene of Thai H4N9 virus (CU-LM1984) and other N9 influenza A viruses**. The phylogenetic tree was generated using the neighbor-joining algorithm. Bootstrap analysis with 1000 replicates and posterior probability from BMCMC analysis were performed for confirming tree topology (Bootstrap, posterior probability). The H4N9 influenza virus characterized in the study is highlighted by triangle.

Phylogenetic analysis was performed using MEGA 4.1 program (Tempe, AZ, USA) with neighbor-joining method with Kimura 2-parameter. Nucleotide sequences of H4N6 (n = 24) and N9 (n = 30) representing different geographic locations, host species and Eurasian/North American lineages were included for phylogenetic analysis. Phylogenetic analysis of the hemagglutinin (HA) gene showed that the H4 gene can be divided into 2 major lineages, the Eurasian and North American, respectively (Figure 1). The HA genes of both H4N6 and H4N9 viruses from Thailand were grouped into the Eurasian lineage. As for the Neuraminidase (NA) gene, phylogenetic analysis of N6 and N9 showed that both NA subtypes also clustered in the Eurasian lineage (Figure 2 and Figure 3). Phylogenetic analyses of the remaining 6 internal genes of the H4N6 and H4N9 influenza viruses were also conducted. The results showed that all internal genes clustered in the Eurasian lineage, similar to both HA and NA genes (Additional file 1 Fig S1). The results from phylogenetic analysis indicated that both H4N6 and H4N9 recovered from Thailand contain all gene segments derived from avian influenza A viruses of the Eurasian lineage. This may be due to the specific non-overlapping flight paths of wild and migratory birds between the Pacific and North American regions.

To genetically analyze the viruses, the nucleotide similarities of each gene of both subtypes H4N6 and H4N9 were generated by using nucleotide BLAST program available at GenBank database. Nucleotide sequences and deduced amino acids of each gene of H4N6 and H4N9 viruses were aligned and compared by using the MegAlign program (DNASTAR). Sequence homologies of each gene of the H4N6 and H4N9 influenza A viruses with highest nucleotide identities to reference influenza A strains are shown in Table 1. The results showed that both H4N6 viruses (CU-LM 1973 and CU-LM 1983) and H4N9 (CU-LM 1984) display the most pronounced nucleotide identities to avian influenza viruses of the Eurasian lineage. The HA gene of all 3 viruses shows the highest nucleotide similarity to A/duck/Taiwan/wb1104/2006(H4N6) at 97%. The NA gene of both N6 and N9 subtypes has the highest nucleotide similarity to A/duck/Eastern China/01/2007 (H4N6) and A/duck/Mongolia/119/2008(H7N9), respectively. H4N6 (CU-LM1983) and H4N9 (CU-LM1984) are highly similar in their HA gene (99.6%) and 6 internal genes (PB2, PB1, PA, NP, NS, M) (98.4-100%), while the NA genes of CU-LM1983(N6) and CU-LM1984(N9) show only 38.1% nucleotide identity (64.6% amino acid identity) because of subtype differences. This finding may suggest that co-infection with H4N6 and H4N9 occurred in the same host. It is possible that the H4N9 virus has resulted from reassortment of the H4N6 backbone and the NA gene (N9) from a separate unknown HA influenza A virus subtype (H?N9).

**Table 1:** Sequence homology of whole genome of three H4 isolates in this study compared to nucleotide sequences available in GenBank database.

a). A/DK/THA/CU-LM1973/09 (H4N6)				
Gene	Position	Virus with the highest percentage of nucleotide identity	Genbank accession #	% nucleotide identity
PB2	1-2239	A/duck/Hokkaido/Vac-3/2007(H5N1)	AB355926.1	98%
PB1	1-2271	A/mallard/Hokkaido/24/2009(H5N1)	AB530990.1	98%
PA	39-2133	A/environment/Dongting Lake/Hunan/3-9/2007(H10N8)	GQ325646.1	99%
HA	1-1679	A/duck/Taiwan/wb1104/2006(H4N6)	GU066565.1	97%
NP	1-1462	A/duck/Thailand/AY-354/2008(H3N2)	FJ802402.1	97%
NA	1-1405	A/duck/Eastern China/01/2007(H4N6)	EU429790.1	97%
M	1-982	A/gull/Astrakhan/1846/1998(H13N6)	GU052229.1	99%
NS	1-817	A/teal/Egypt/912908/2005(H10N7)	EU599315.1	98%
**b) A/Dk/THA/CU-LM1983/09 (H4N6)**				

**Gene**	**Position**	**Virus with the highest percentage of nucleotide identity**	**Genbank accession #**	**% nucleotide identity**

PB2	1-2211	A/migratory duck/Hong Kong/MP206/2004(H5N2)	EF597476.1	97%
PB1	1-2265	A/mallard/Hokkaido/24/2009(H5N1)	AB530990.1	98%
PA	9-2133	A/environment/Dongting Lake/Hunan/3-9/2007(H10N8)	GQ325646.1	98%
HA	1-1682	A/duck/Taiwan/wb1104/2006(H4N6)	GU066565.1	97%
NP	1-1462	A/duck/Thailand/AY-354/2008(H3N2)	FJ802402.1	98%
NA	1-1406	A/duck/Eastern China/01/2007(H4N6)	EU429790.1	97%
M	1-982	A/gull/Astrakhan/1846/1998(H13N6)	GU052229.1	99%
NS	1-821	A/teal/Egypt/912908/2005(H10N7)	EU599315.1	99%
**c) A/Dk/THA/CU-LM1984/09 (H4N9)**				

**Gene**	**Position**	**Virus with the highest percentage of nucleotide identity**	**Genbank accession #**	**% nucleotide identity**

PB2	55-2212	A/migratory duck/Hong Kong/MP206/2004(H5N2)	EF597476.1	97%
PB1	5-2271	A/mallard/Hokkaido/24/2009(H5N1)	AB530990.1	98%
PA	9-2133	A/environment/Dongting Lake/Hunan/3-9/2007(H10N8)	GQ325646.1	99%
HA	1-1684	A/duck/Taiwan/wb1104/2006(H4N6)	GU066565.1	97%
NP	1-1470	A/duck/Thailand/AY-354/2008(H3N2)	FJ802402.1	98%
NA	24-1377	A/duck/Mongolia/119/2008(H7N9)	AB481213.1	97%
M	1-955	A/gull/Astrakhan/1846/1998(H13N6)	GU052229.1	99%
NS	1-818	A/teal/Egypt/912908/2005(H10N7)	EU599315.1	99%

**Figure 4:**
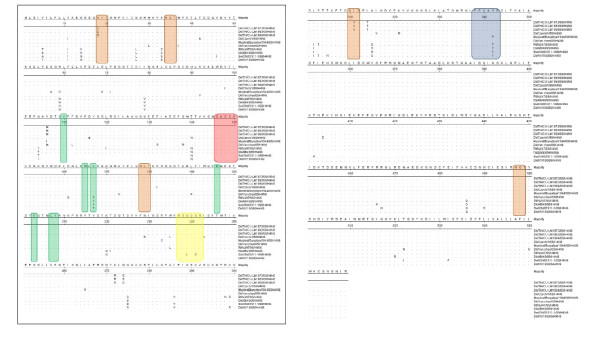
**Comparison of deduced amino acid sequences of H4N6 and H4N9 using MegAlign program**. Identical amino acids are shown as dots. Cleavage site, N-linked glycosylation site, receptor binding site, left edge and right edge of receptor-binding pocket are shown in boxes.

The deduced amino acids of the H4 gene of influenza A isolates were aligned and compared with those of reference H4N6 and H4N9 viruses representing the Eurasian and North American lineages published at the GenBank database using the MegAlign program. Our result showed that amino acids at the HA cleavage site in the Thai H4N6 and H4N9 viruses are "PEKASR", similar to most avian isolates in the Eurasian lineage, while those of the North American lineage including ruddy turnstone (NJ/47/85), mallard (ALB/49/95) and swine (ONT/1911-1/99) isolates are "PEKATR" (Table 2)[13] The HA cleavage site pattern of both Thai H4N6 and H4N9 indicates low pathogenic characteristics of influenza A viruses. It has been known that addition of multiple amino acids at the cleavage site such as Arginine (R) and Lysine (K) may turn LPAI into HPAI [14]. However, to further confirm virus pathogenicity, an intravenous pathogenicity index test (IVPI) should be performed [15]. Amino acids at receptor binding sites (HA98,153,155,183,190, 194,195 (H3 numbering system)) were also analyzed (Table 2). In this study, the amino acids of Thai H4N6 and H4N9 at positions 224-229 and 134-138 were "RGQSGR" and "GKSGA", respectively. It is noteworthy that the receptor binding sites of H4N6 and H4N9 especially, Q226 and G228 are similar to all H4N6 in both Eurasian and North American lineages suggesting that these viruses would preferentially bind to 2,3 linked sialic acid receptors predominant in avian species [16]. One H4N6 virus from swine (A/Swine/Ontario/01911-1/99) contains amino acid changes at positions 226 (Q226L) and 228 (G228S) indicating high affinity of the virus to SA 2,6Gal receptors which are dominant in mammalian species [13,16]. N-linked glycosylation sites of the H4 gene were also analyzed. Three N-linked glycosylation sites in HA1 and one glycosylation site in HA2 were observed in Thai H4 viruses (Figure 4). It should be noted that one glycosylation site in HA1 of Thai H4 influenza A isolates was absent due to the amino acid changes N2D resulting in loss of glycosylation ability [17]. Thai H4N6 and H4N9 contain less N-linked glycosylation sites than the viruses described in a previous report [17]. Previous studies reported that an absent of N-liked glycosylation site of HA protein would altered biological functions of the virus [18,19].

**Table 2:** Genetic analysis of amino acids at HA cleavage site and receptor binding sites of H4 gene of three H4 isolates in this study and reference H4N6 and H4N9 virus

	HA cleavage	Receptor-binding site						
	
H3 numbering system*	320-329	98	153	155	183	190	194	195
H4 position	338-343	110	165	167	196	203	207	208
A/Dk/TH/CU-LM1973/09 (H4N6)	PEKASR	Y	W	V	H	E	L	Y
A/Dk/TH/CU-LM1983/09 (H4N6)	PEKASR	Y	W	V	H	E	L	Y
A/Dk/TH/CU-LM1984/09 (H4N9)	PEKASR	Y	W	V	H	E	L	Y
A/Dk/Czech/1/56 (H4N6)	PEKASR	Y	W	V	H	E	L	Y
A/Muskrat/Buryatiya/1944/00 (H4N6)	PEKAPR	Y	W	V	H	E	L	Y
A/mallard/Yan Chen/2005 (H4N6	PEKASR	Y	W	V	H	E	L	Y
A/turnstone/NJ/47/85 (H4N6)	PEKATR	Y	W	T	H	E	L	Y
A/mallard/Alberta/49/1995(H4N6)	PEKATR	Y	W	T	H	E	L	Y
A/Sw/ON/01911-1/99 (H4N6)	PEKATR	Y	W	T	H	E	L	Y
A/Dk/NY/180/86 (H4N9)	PEKATR	Y	W	T	H	E	L	Y
								
		Left edge of receptor-binding site						
		
H3 numbering system		224	225	226	227	228	229	
H4 position		237	238	239	240	241	242	
	
A/Dk/TH/CU-LM1973/09 (H4N6)		R	G	Q	S	G	R	
A/Dk/TH/CU-LM1983/09 (H4N6)		R	G	Q	S	G	R	
A/Dk/TH/CU-LM1984/09 (H4N9)		R	G	Q	S	G	R	
A/Dk/Czech/1/56 (H4N6)		R	G	Q	S	G	R	
A/Muskrat/Buryatiya/1944/00 (H4N6)		R	G	Q	S	G	R	
A/mallard/Yan Chen/2005 (H4N6		R	G	Q	S	G	R	
A/turnstone/NJ/47/85 (H4N6)		R	G	Q	S	G	R	
A/mallard/Alberta/49/1995(H4N6)		R	G	Q	S	G	R	
A/Sw/ON/01911-1/99 (H4N6)		R	G	L	S	S	R	
A/Dk/NY/180/86 (H4N9)		R	G	Q	S	G	R	
								
		Right edge of receptor-binding site						
		
H3 numbering system		134	135	136	137	138		
H4 position		146	147	148	149	150		
	
A/Dk/TH/CU-LM1973/09 (H4N6)		G	K	S	G	A		
A/Dk/TH/CU-LM1983/09 (H4N6)		G	K	S	G	A		
A/Dk/TH/CU-LM1984/09 (H4N9)		G	K	S	G	A		
A/Dk/Czech/1/56 (H4N6)		G	K	S	G	A		
A/Muskrat/Buryatiya/1944/00 (H4N6)		G	K	S	G	A		
A/mallard/Yan Chen/2005 (H4N6		G	K	S	G	A		
A/turnstone/NJ/47/85 (H4N6)		G	K	S	G	A		
A/mallard/Alberta/49/1995(H4N6)		G	K	S	G	A		
A/Sw/ON/01911-1/99 (H4N6)		G	K	S	G	A		
A/Dk/NY/180/86 (H4N9)		G	K	S	G	A		

The influenza A virus subtypes H4N6 and H4N9 found in LBMs can be a potential risk to birds and humans. The viruses were shed in feces and contaminated the wire stack cages in LBM. This setting provides an appropriate condition for virus transmission among birds kept in the cage nearby or below [20]. Consequently, a small outbreak of influenza A viruses H4N6 and H4N9 in the birds in LBM can occur. Fortunately, the H4N6 and H4N9 viruses circulating in this LBM are of low virulence and hardly capable of infecting humans. However, if HPAI or newly reassorted viruses with high virulence circulate in the markets in the future, this would pose a threat to vendors, consumers and people who enter the markets. For example, in China, HPAI H5N1 infection was reported in one patient who had visited LBMs. The study showed that LBM can be a potential source of HPAI in a local community and infection of humans can occur not only by direct but also by indirect contact [4].

The public should be made aware of the significance of the influenza viruses, the hosts, the environment and potential virus reassortment especially in LBMs. Examples of influenza prevention and control strategies in the LBM include 1) creating a rest day to interrupt the LBM's flow system, which could significantly reduce the occurrence of the virus in LBMs [21], 2) disinfection of fomites in LBMs as the presence of influenza A virus in fomites has been documented [22], 3) educational campaigns, vendors should be informed on how to protect themselves from influenza A virus infection. Since surveillance of influenza A virus in LBMs can serve as a good early warning system [6], influenza A viruses isolated in LBMs can reflect the status and the diversity of the viruses circulating in local communities. Continuous influenza A virus surveillance in LBMs should be performed in order to understand the epidemiology of influenza A virus in LBMs and to monitor influenza virus evolution.

In conclusion, influenza A virus subtypes H4N6 and H4N9 were isolated from ducks in a live bird market in Bangkok, Thailand. Both H4N6 and H4N9 subtypes recovered from ducks had never been reported in Thailand. Whole genome sequences of the viruses were determined and submitted to the respective databases. Phylogenetic analysis of H4N6 and H4N9 viruses showed that gene segments of the viruses were derived from the Eurasian linage. Genetic analysis of the viruses also showed that the viruses display low pathogenic characteristics, with amino acids specific to avian influenza viruses.

## Competing interests

The authors declare that they have no competing interests.

## Authors' contributions

TW conducted sample collection, virus isolation, virus identification, genome sequencing, phylogenetic analysis and drafted the manuscript. MW, SB, NB participated in sample collection, virus isolation and whole genome sequencing. PK helped designed LBM surveillance, data analyses. AA designed LBM surveillance, data analyses and final approval of the manuscript. All authors read and approved the final manuscript.

## Supplementary Material

Additional file 1 **Figure S1**. Phylogenetic analysis of internal genes of Thai H4N6 and H4N9 viruses and other H4N6 and H4N9 influenza A viruses. The phylogenetic tree was generated using the neighbor-joining algorithm. Bootstrap analysis with 1000 replicates and posterior probability from BMCMC analysis were performed for confirming tree topology (Bootstrap, posterior probability). The H4N6 and H4N9 influenza viruses characterized in the study are present in triangle. Click here for file
