# Supplementary figures and images for: Genetic characterization of avian influenza subtype H4N6 and H4N9 from live bird market, Thailand
Source: Virol J. 2011 Mar 21;8:131. doi: 10.1186/1743-422X-8-131 (PMC3071790; doi:10.1186/1743-422X-8-131)

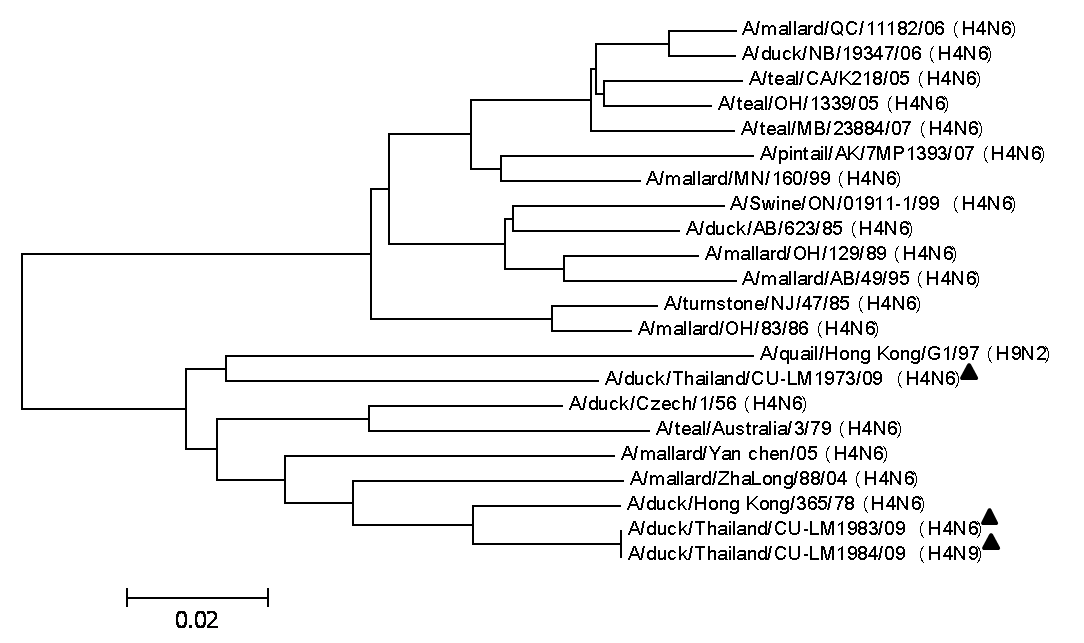


**PB2**


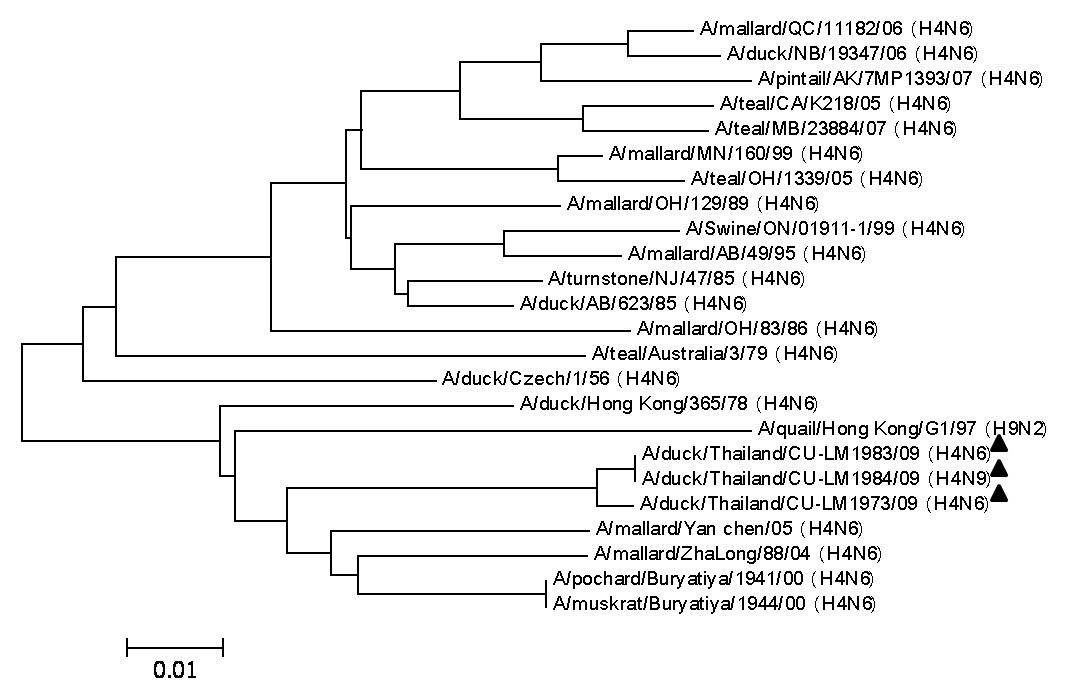


**PB1**


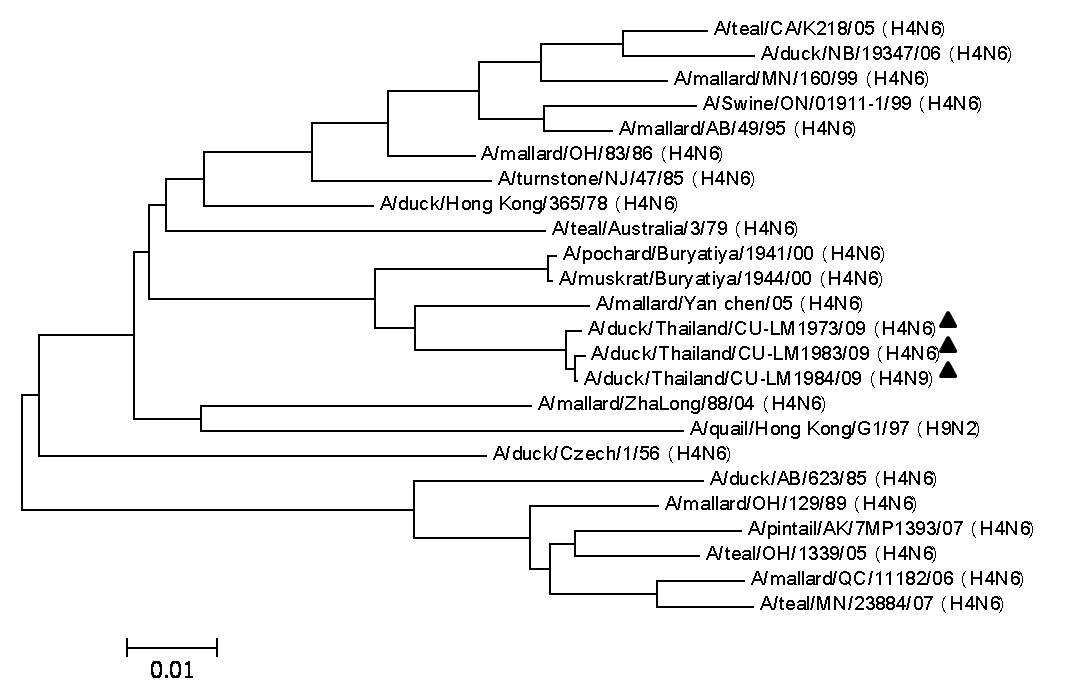


**PA**


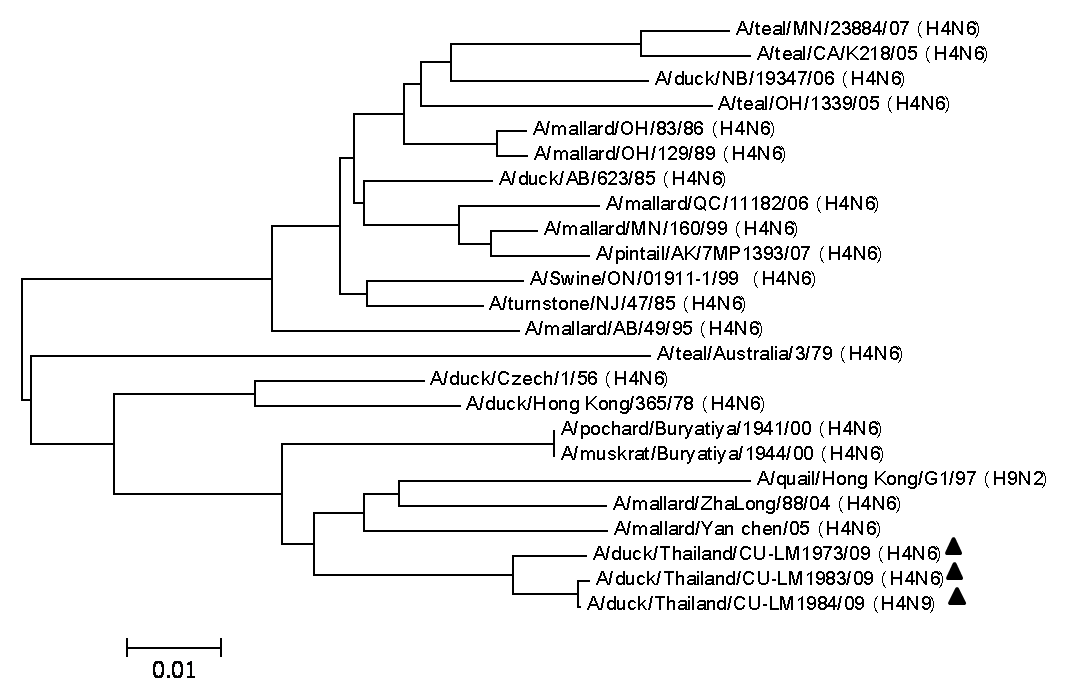


**NP**


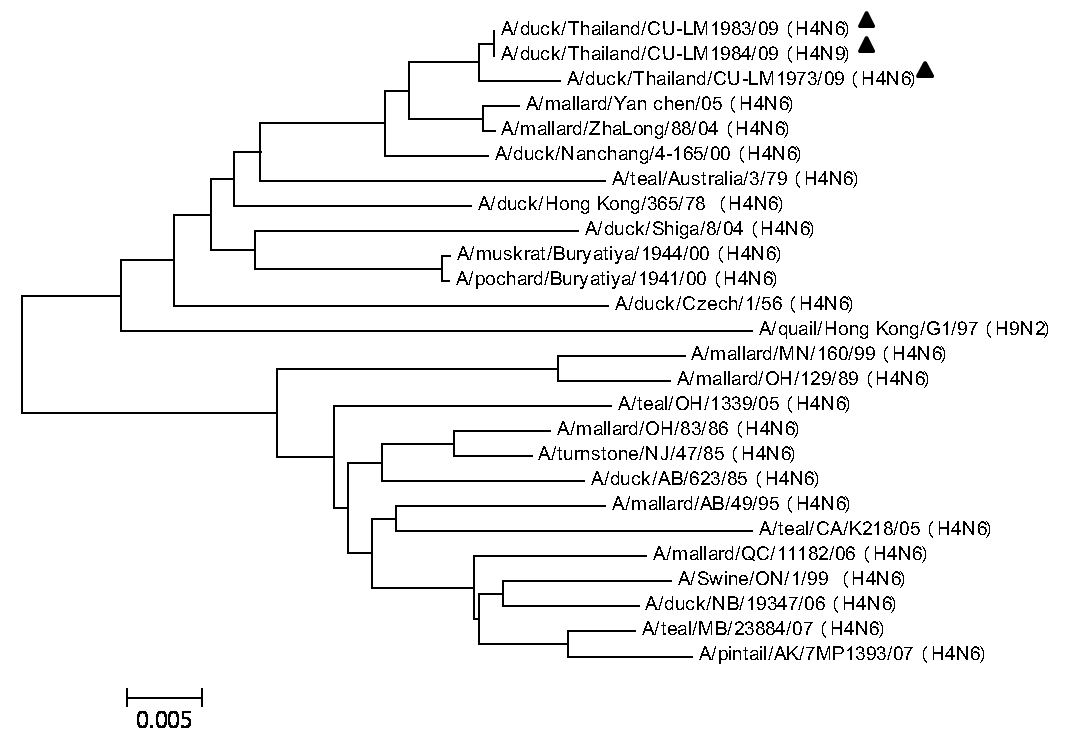


**M**


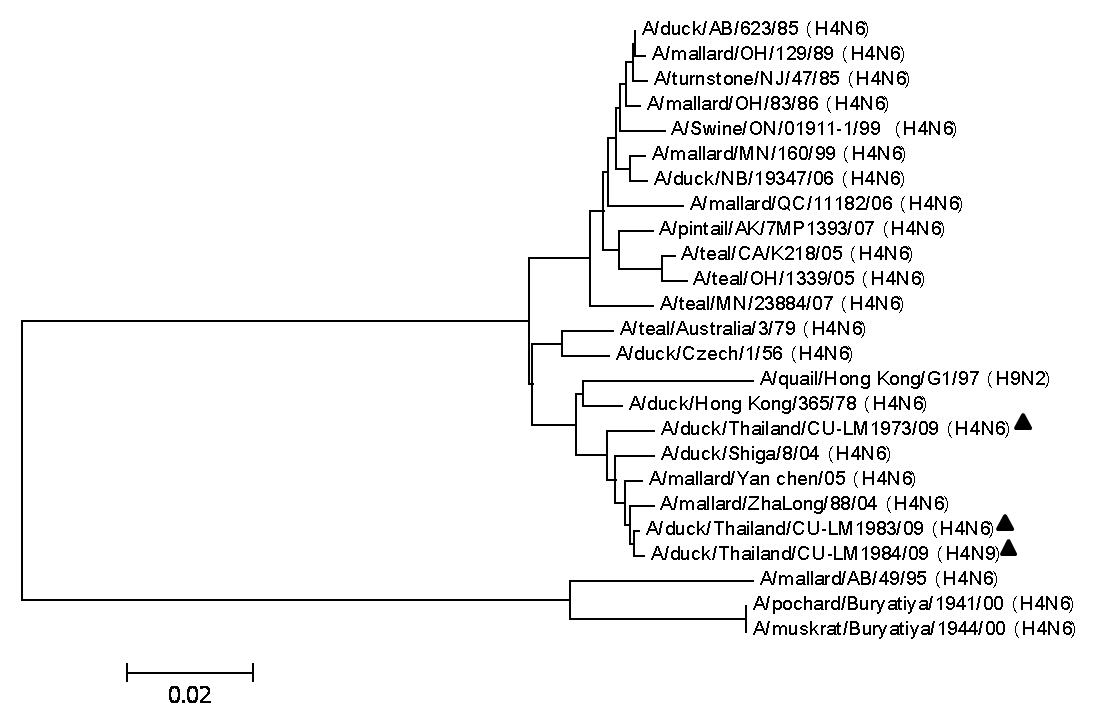


**NS**

Supplement: Additional file 1 — Figure S1. Phylogenetic analysis of internal genes of Thai H4N6 and H4N9 viruses and other H4N6 and H4N9 influenza A viruses. The phylogenetic tree was generated using the neighbor-joining algorithm. Bootstrap analysis with 1000 replicates and posterior probability from BMCMC analysis were performed for confirming tree topology (Bootstrap, posterior probability). The H4N6 and H4N9 influenza viruses characterized in the study are present in triangle. [file 1743-422X-8-131-S1.DOC]
